# Protein-Polymer Matrix Mediated Synthesis of Silver Nanoparticles

**DOI:** 10.5772/59297

**Published:** 2014-01-01

**Authors:** Swati Mishra, Suprabha Nayar

**Affiliations:** 1 Materials Science & Technology Division, CSIR-National Metallurgical Laboratory, Burmamines, Jamshedpur, India

**Keywords:** Silver Nanoparticles, Protein-polymer Matrix, Photoluminescence, Drug Delivery

## Abstract

Silver nanoparticles were synthesized in the protein-polymer matrices of two different ratios to obtain a stringent control over the morphology. UV-visible spectrophotometry showed a single plasmon resonance peak at 416nm and 418nm respectively, confirming the formation of silver nanoparticles. X-ray diffractometry confirmed that the peaks matched with that of the reference silver. Both confocal microscopy and FEG-SEM confirmed the uniform morphology of the synthesized particles dependent on the template ratio. Doubling the protein-polymer concentration results in greater stability, more nucleation sites and hence restricted growth. Photoluminescence of the sample in the doubled matrix was found to be much greater at any given wavelength, meaning the flexibility and rigidity of interacting molecules affects the luminescence signal. The interaction in turn is dependent on the proximity of the proteins and polymer in the dispersion that forms a template and dictates the synthesis.

## 1. Introduction

Organic-inorganic nanocomposites have attracted immense attention because of their potential to combine the features of polymeric materials with those of inorganic materials. Colloidal silver nanoparticles (AgNPs) synthesized in a protein-polymer matrix have numerous applications as biosensors, antimicrobial agents, catalysts and as new generation light weight electronic devices [1–2]. A battery of techniques are available in the literature to synthesize AgNPs in aqueous as well as in non-aqueous, media [3-9]. The general philosophy of controlled synthesis of metal nanoparticles from its salt solution is based on designing the right template, templating agents in addition to controlling morphology and also preventing the nanoparticles from agglomeration [10-12]. Examples include the work of Rita et al. [13], who reported the synthesis of AgNPs using hydroquinone and sodium citrate as the reducing agent with neutral polymers poly (vinyl-pyrrolidone) (PVP) and poly (vinyl alcohol) (PVA) as stabilizers. Soloman et al. [14] synthesized AgNPs by reducing silver nitrate with sodium borohydride without using any surfactant leading to aggregation. Kim et al. [15] chose various silver salts as the starting material and examined the effect of an initial precursor on the rate of nanoparticle formation. They found that by using silver salts, such as silver tetrafluoroborate (AgBF_4_), silver hexafluorophosphate (AgPF_6_) and silver perchlorate (AgClO_4_), the reaction proceeded rapidly in the beginning and then slowed, whereas in the case of silver nitrate (AgNO_3_) the reaction rate was slower but constant. Prasad and co-workers [16] used sophorolipids for the synthesis and stabilization of AgNPs. In comparison to *in situ* reduction of silver ions in aqueous solution, a polymer matrix-mediated reduction of silver ions has been found to be more suitable for the synthesis of polymer-silver nanocomposite particles for various biomedical applications [17]. Matrix mediated synthesis of metal nanoparticles, a derivative of biomineralization termed “biomimetic synthesis”, yields nanoparticles with stringent control over shape and size [18,19]. Various groups have reported *in situ* synthesis of AgNPs in PVA with and without another reducing agent [20-22]. Clemensen et al. studied the effect of silver ion concentration on PVA mediated synthesis of AgNPs [23]. Here we have used a combination of PVA (P), collagen (C) and Bovine Serum Albumin (BSA) (B) in two different concentrations for the synthesis. The idea of using three ligands is to improve stability and enhance functionality; the mutual interaction of the protein-polymer is dependent on their concentration and we have optimized a certain combination for our reactions. We have reported the usage of the same combination for the synthesis of ferrofluids and magnetic hydroxyapatite [24–25]. “In situ synthesis of hydroxyapatite on fluidic IONPs in ambient condition” communicated to, *J. Mater. Sci.-Mater. Med* To the best of our knowledge, no one else has used the above mentioned combination for the synthesis of AgNPs that also plays a dual role of a template and a stabilizer.

## 2. Material and Methods

### 2.1 Materials

AgNO3 was purchased from Nice chemicals Ltd. and PVA(P) (MW 95000, degree of hydrolysis 90%) from Acros Organics, sodium hydroxide and dextrose from MERCK, Mumbai, India, Collagen (C) from SIGMA ALDRICH, St Louis, USA; BSA(B)-fraction V (bovine serum albumin) from ACROS ORGANICS, New Jersey, USA. All the chemicals were of analytical grade and used without further purification. Double distilled water was used throughout the experiment.

### 2.2 Synthesis of Ag CPB

C 0.01%, P 0.01% and B 0.001% were dissolved in 20ml (each separately) in Phosphate Buffer Saline and then mixed together and allowed to equilibrate for six hours. 2mM AgNO3 solution was prepared in 40ml double distilled water. AgNO_3_ solution was added to the above mixture, the volume was made up to 100ml. After one and a half hours of stirring at 300rpm, 2ml of 4M NaOH was added drop wise to the sample in stirring condition and the colour change was observed from colourless to a brown colour, due to formation of silver oxide at pH 11.5. 1ml of 1M dextrose was added to the mixture and a dark grey colour was formed due to the reduction of silver oxide to silver. For the synthesis of Ag CPB D the concentrations were doubled to C 0.02%, P 0.02% and B 0.002% keeping other parameters and steps the same as in the synthesis of Ag CPB. The synthesized nanofluids were centrifuged and dialyzed using cellulose membrane, HIMEDIA, Mumbai, India, against double distilled water to remove the by-products and the dialyzed fluid was used for characterization.

### 2.3 Characterization

The visible light absorption of the samples was recorded using a Cary 50 Bio UV-Vis spectrophotometer by VARIAN. Phase identification of the samples was conducted at room temperature x-ray diffraction in D8 DISCOVER BRUKER Diffractometer, AXS GmbH, Germany, operated at 40KV with CuK_α_ radiation (λ=1.5418 Å) within a scanning range of 20°-90° (2⊖) at a step size of 0.02°/step and scanning speed of 1sec/step. The shape and morphology of the nanoparticles were recorded in FEG SEM Nova Nano SEM 430 operated at 15 kV. Images of liquid samples were recorded using a ZEISS CLSM 700 confocal microscope in both differential interference contrast (DIC) and fluorescent mode with no fluorophores. A drop of sample was sandwiched with cover slips on glass micro slides and observed under the microscope. Hydrodynamic diameter (D_H_), zeta potential and polydispersity index (PDI) was measured by a zetasizer DLS NANO 100, MALVERN INSTRUMENTS, USA. The hydrodynamic radius (R_H_ = D_H_/2) was calculated from the diffusion coefficient using the Stokes-Einstein equation,

D=KT/f=KT/6πηRH

Where, K is the Boltzmann constant, T is the temperature, η is the medium viscosity and f=6πηR_H_ is the frictional coefficient for a hard sphere in a viscous medium. The data was measured using the back scattering technique at an angle of 173°. Photoluminescence was recorded using a F-4500 fluorescence spectrophotometer from HITACHI. Drug binding and release were studied using the drug methotrexate, a drug used in the therapy of cancer.

## 3. Results and Discussions

### 3.1 UV-vis Spectrophotometry and X-ray diffractometry

**Figure 1.**
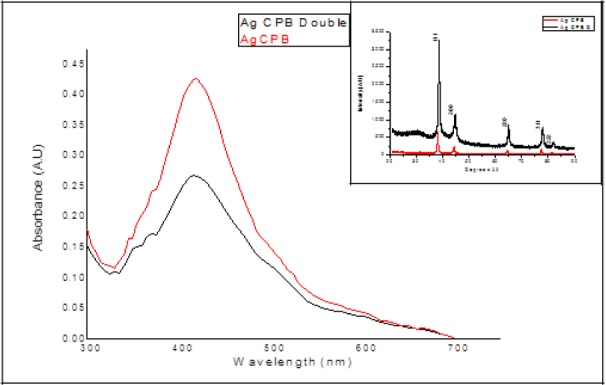
UV-Vis spectroscopy of the Ag CPB and Ag CPB D shows peaks at 418nm and416nm respectively. Inset shows the XRD peaks of both that confirm the formation of AgNPs

UV visible spectra of samples were recorded in the range of 300 to 700nm as shown in Figure 1, post seven times dilution with DDW (double distilled water). There was only one characteristic surface plasmon absorption peak at 416nm for Ag CPB D and at 418nm for Ag CPB. According to Mie's theory anisotropic particles show two or three bands dependent on their shape, the fact that there is only one distinct peak proves a stringent control over nucleation and subsequent growth. The inset shows XRD pattern of both nanofluids indexed w.r.t. standard silver (JCPDS card no. 87-0720). Characteristic peaks corresponding to the Bragg planes (111), (200), (220), (311), (222) confirm the formation of silver nanoparticles. The crystallite size of Ag CPB was 46.95 nm and that of Ag CPB D was 21.16 nm corresponding to the 100% peak, on doubling the concentration of the protein and polymer the size of the nanoparticles were reduced with a slight shift in every peak.

### 3.2 Confocal Microscopy and FEG SEM

**Figure 2.**
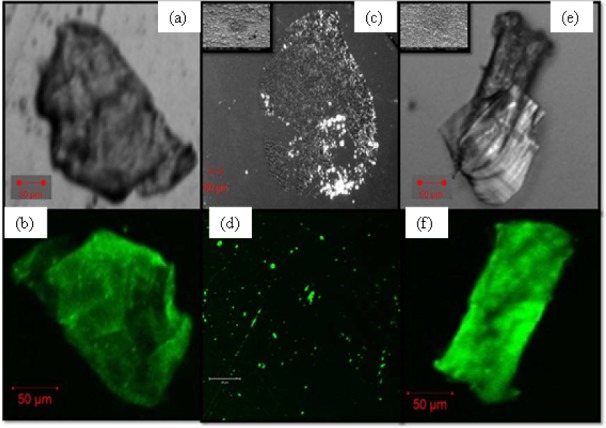
shows the confocal images in differential interference contrast (DIC) mode and fluorescence mode (a), (b) control (c) and (d) Ag CPB, (e) and (f) Ag CPB D. The inset of fig (c) and (e) shows the FEG SEM image of AgNPs, spherical in shape for both.

The final morphology and crystallinity of the nanofluid is dependent on the concentration of the protein and polymer added. The functional groups like amine-(NH_2_), carboxyl (–COOH), and hydroxyl (-OH) not only stabilizes the colloid, but also increases the number of nucleation sites. The matrix of polymer PVA and the two proteins BSA and Collagen forms a well-organized matrix with specific nucleation sites for AgNPs seen in the confocal differential interference contrast (DIC) image. This is similar to the biomineralization process where controlled growth of inorganic component takes place inside an organic matrix [25] as is evident from Figure 2.

The multitude of non-covalent interactions between the organic and inorganic network results in uniform morphology, spherical nanoparticles were seen in FEG SEM in both the samples. The size only reduced due to an increase in nucleation sites with the doubling of the matrix constituents. Green auto fluorescence was observed corresponding to an Alexa Flour AF488 filter in all the samples in confocal micrographs. Fluorescence at certain defined positions clearly shows site-specific nucleation in both, Ag CPB at greater distances as compared to the close tight fit of Ag CPB D that reduces size.

### 3.3 Drug binding and release and dynamic light scattering

**Figure 3.**
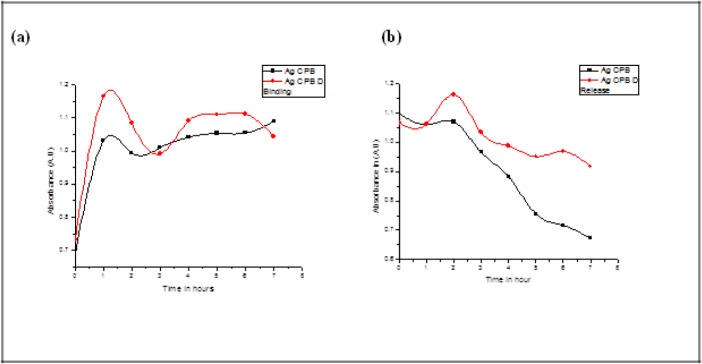
The above graph depicts the time versus absorbance for the binding and release of the drug methotrexate to the sample Ag CPB in black and Ag CPB D in red

Drug binding and release of the samples, using the Methotrexate drug, was studied by measuring absorbance at 305nm. The absorbance was recorded every hour for a total period of seven hours both for binding and post dialysis using 0.05% NaCl for release (Ag CPB nanoparticles do not cross the dialysis membrane while methotrexate does so). Both samples have equally good binding properties with the drug but when the release profile was studied it was found that Ag CPB D releases the drug in a more controlled way compared to Ag CPB, which shows immediate release of the drug as seen in Figure 3. The entrapment efficiency for Ag CPB and Ag CPB D was found to be 80.4% and 86%, respectively calculated using:

$$\begin{array}{r} \%\;\text{Entrapment}\;\text{efficiency}=\underline{\text{Amount}\;\text{of}\;\text{drug}\;\text{loaded}}\times 100\\ \text{Initial}\;\text{amount}\;\text{of}\;\text{drug}\;\text{added}\;\text{into}\;\text{the}\;\text{system} \end{array}$$

This shows that doubling the template concentration does not necessarily double the entrapment. That Ag CPB D has a controlled release profile is an indicator that it has the potential of sustained release of the drug, which is a requirement in the release of cancer drugs. These two formulations of nanofluids can be used for two different purposes; Ag CPB D for sustained therapy and Ag CPB where the drug is needed quickly.

The D_H_ of Ag CPB was found to be 70.26nm, zeta potential −28.7mV and PDI 0.259 as compared to 92.04nm, −11.9mV and 0.207 of Ag CPB D. These fluids are stabilized with the protein-polymer interaction, but there is a certain optimum ratio of the proteins and the polymer that is needed for the best stability and CPB seems to be better than CPB D in terms of stability. As seen from FEGSEM there is fusion of the uniform-sized globules approximately 100nm in size, when one doubles the protein-polymer concentration and that could be the reason for the lesser stability of the fluids. These results are in good agreement with the UV-visible results. The hydrodynamic size increases with a double CPB concentration, because of the increased bonding possibilities with water molecules.

### 3.4 Photoluminescence

**Figure 4.**
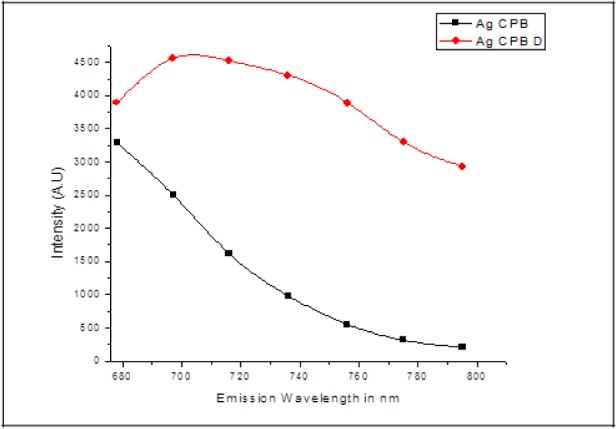
Comparison of the emission intensity variation for Ag CPB and Ag CPB D in the wavelength range of 340 to 400nm in luminescence mode

The two samples Ag CPB and Ag CPB D were excited in the range of 340nm to 400nm and photoluminescence emission was recorded and plotted against intensity for both samples. Luminescence of Ag CPB D was found to be almost thrice compared to the Ag CPB at the same wavelength. The nature of the chemical structure of a molecule in terms of flexibility and rigidity is of major influence on the luminescence signal. Molecules that have a high degree of flexibility will tend to decrease luminescence due to higher collisional probability. Rigid structures have lower probability of collisions and thus have more luminescence potential as seen in Figure 4. When we double the protein-polymer concentration, we are essentially increasing the rigidity and hence these fluids may prove important as probes.

## 4. Conclusions

The *in situ* reduction of silver ions in protein-polymer matrix is an attractive process having industrial potential as it is easily scalable. The mechanism of synthesis of silver nanoparticles using PVA with collagen and with BSA, individually or in combination, is well documented in the literature. Previous, studies with only PVA shows that its concentration (which is usually quite high), plays a major role in determining the dimensions, as well as the stability of the silver colloidal solution. This new combination of the matrix has only been reported by our group. The results prove that the Ag CPB D and Ag CPB though both show stability and superior characteristics, proving that in addition to ferrofluids and magnetic hydroxyapatite, the matrix is also good for Ag nanoparticles. This study shows that using a combination of proteins and polymer we can not only decrease the total concentration of the organic content but also increase multi-functionality for applications.
